# Culture and Happiness

**DOI:** 10.1007/s11205-014-0747-y

**Published:** 2014-09-06

**Authors:** Dezhu Ye, Yew-Kwang Ng, Yujun Lian

**Affiliations:** 1 Department of Finance, Research Institute of Finance, Jinan University, Guangzhou, China; 2 Division of Economics, Nanyang Technological University, Singapore, 637332 Singapore; 3 Emeritus Professor, Monash University, Melbourne, Australia; 4 Department of Finance, Lingnan College, Sun Yat-Sen University, Guangzhou, China

**Keywords:** Happiness, Subjective well-being, Culture, Power distance, Gender egalitarianism, GLOBE

## Abstract

Culture is an important factor affecting happiness. This paper examines the predictive power of cultural factors on the cross-country differences in happiness and explores how different dimensions of cultural indices differ in their effects on happiness. Our empirical results show that the global leadership and organizational behavior effectiveness nine culture indices are all significantly related with happiness. Out of these nine indices, power distance (PDI) and gender egalitarianism (GEI) play the most important and stable role in determining subjective well-being (SWB). We further examine the relative importance of the various variables in contributing to the R-squared of the regression. The results show that PDI is the most important, accounting for 50 % of the contributions to R-squared of all variables, or equalling the combined contributions of income, population density and four other traditional variables. The contribution of GEI is 37.1 %, also well surpassing other variables. Our results remain robust even taking account of the different data for culture and SWB.

## Introduction

International surveys of subjective well-being (SWB) show consistent mean level differences across nations (e.g., Inglehart and Klingemann 2000; Veenhoven 1993). Inglehart and Klingemann (2000) compare several waves of SWB at the country level from the World Values Survey (WVS) and discover substantial cross-country differences which persist and are stable. For example, in the 1998 survey, 65 % of Danes were very satisfied with their lives, while only 5 % of the Portuguese said they were very satisfied. In the several surveys before that, the proportion of Danes who were very satisfied with life was also around 12 times that of the Portuguese. Kenny (1999) also finds that the variance in the happiness index of a single country in different periods is far less than the variance in the happiness index of different countries in the same periods. This suggests that there may exist significant country fixed effects in cross-country comparison of SWB. Explaining cross-country differences in SWB is thus an important issue (Diener et al. 1995; Diener 2000; Kenny 1999; Inglehart and Klingemann 2000; Heukamp and Arino 2011). This paper uses the SWB data from WVS and the culture data from the GLOBE (Global Leadership and Organizational Behavior Effectiveness) culture project to investigate the effects of culture on SWB, focusing on comparing the explanatory power of culture variables relative to traditional factors, and the relative importance of different culture variables in explaining the differences of SWB between countries.

In the literature, assessments of well-being sometimes use questions that refer to “life satisfaction”, sometimes to “happiness”, and sometimes to the overall subjective well-being (SWB). No matter which format or wording is used, the findings tend to be similar (Layard 2005). As in Heukamp and Arino (2011), our empirical basis is the SWB data from WVS. Therefore, strictly speaking, we deal with SWB. However, partly due to the similarity in empirical results and partly due to the ease of discussion, we use SWB and happiness largely interchangeably.

Hofstede and Bond (1988) define culture as “the collective programming of the mind that distinguishes the members of one category of people from those of another”, and culture can be transferred largely unchanged from generation to generation (Guiso et al. 2006). The stable cross-country differences in culture and the stable cross-country differences in SWB are very similar. Thus, it is possible that the differences in culture may be closely related with the levels of SWB in different countries.

A number of culture-oriented psychologists have emphasized the critical role of public meanings (folk theories and common sense) and practices (daily routines) that, taken as a whole, define a culture by shaping emotions (Benson 2000; Bruner 1990; Kitayama 2002; Markus and Kitayama 1991; Shweder and Sullivan 1993). In other words, emotions and cognitions are always situated and embedded in specific cultural contexts. Culture may affect happiness in terms of the amount, extent, or degree of happiness. Thus, culture “can influence mean levels of SWB” (Diener et al. 2003, p. 406).

For example, a culture of individualism prevails in Western countries in Europe and America. People emphasize individual freedom, individual achievement, and the pursuit of individual positive feelings. Thus, the relationships between SWB and individual effort and achievement are more direct, possibly making happiness levels higher. In the collectivist culture zones including Japan, Korea, and China, people put relatively more emphasis on human relationships, including families, colleagues and neighbours. Happiness feelings are affected relatively more by the evaluation of others. The relationships between SWB and individual effort and achievement are not clear. This may make their happiness levels lower than in the individualist countries. According to the WVS survey in 2006, out of 50 countries, Colombia ranked first in life satisfaction, Denmark second, Japan 25th, and Korea 35th. Despite their wealth and high levels of economic development and welfare facilities, especially relative to the Latin American countries, Japan and Korea, which belong to the culture that emphasizes relationship, ranking very low in life satisfaction.

The impact of a country’s culture on SWB has been explained through different culture-dependent definitions of achievement. As external environmental factors have rather small influences on SWB (e.g., social–economic factors can only explain 6 % of the variation in SWB, Inglehart and Klingemann 2000), researchers shifted to examining the top-down social and psychological factors that affect how individuals perceive external events and environments. One of the main variables is culture. Inglehart and Klingemann (2000) note that, while within any given country, genetic factors account for most of the variance in well-being, for the much larger variation in well-being across different countries, cultural differences explain at least as much as genetic factors. Similarly, Diener et al. (2003) believe that personality explains differences in SWB between individuals, while culture explains those between countries.

In specific discussion on the influence of culture on SWB, it is common to divide culture into two large groups, one consisting of European-American cultures and the other of East-Asian cultures, and examine the differences between these two groups in terms of the meanings of happiness, the motivations underlying happiness, and the predictors of happiness (Uchida et al. 2004). A basic conclusion is that people in countries with an individualist culture have higher levels of SWB than those in a collectivist culture; this may partly explain the East-Asian happiness gap (Ng 2002). Oishi et al. (1999) and Inglehart et al. (2008) point out that people in countries with individualist cultures have more freedom of choice, which correlates positively with SWB. Suh (2002) suggests that in countries with individualist cultures, people have stronger self-identity consistency, a more consistent self-view. This means that they have clearer self-knowledge, and, most notably, they have self-experiences that are less affected by the perspectives of others. Hence, it is easier for them to have direct experience of happiness. Kwan et al. (1997) point out that people in countries with individualist cultures have stronger self-esteem, raising their feeling of happiness. In summarizing, the different influences on happiness for East-Asian and Euro-American cultures, Lu et al. (2001) regard the East-Asians as socially oriented and the Euro-Americans as individually oriented. Schimmack et al. (2002) point out that the middle-of-the-road attitude towards happiness and unhappiness of the East-Asians may be rooted in the dialectical thinking in Asian philosophies (such as Buddhism and Daoism), while Europeans and Latin Americans are more inclined towards the direct feelings of happiness.

The contributions discussed above regarding the relationship between culture and cross-nation differences in SWB significantly increase our understanding of SWB. However, there are still some inadequacies.

Firstly, the proxy variables used to measure culture are dummy variables, e.g., country dummy variables and religion dummies (Heukamp and Arino 2011), languages (Diener et al. 2003), and the individualist-collectivist dummy (Schyns 1998; Lu and Gilmour 2004; Uchida et al. 2004).^1^ As a measurement of culture, the number of dimensions is too small. Measures such as religion and language dummies can only provide one dimension of culture. Moreover, the country dummies may provide biased measures of culture, because the country dummy variables can only explain country individual effects, while these effects are not only caused by cultural factors but also other factors. For example, apart from culture, factors such as geography and climate do not change much over time and they may also cause country fixed effects. Secondly, there is no quantitative measurement of country fixed effects in cross-country differences in SWB. Many papers recognize the existence of these country fixed effects, but give no necessary quantitative explanations. Thirdly, there may exist an endogeneity problem between culture and happiness. This has not been discussed adequately in the literature. Fourthly, as the content of culture is very rich, the question as to which dimension of culture may affect happiness most has not been explored adequately.

In this paper, we use some well-accepted national culture indices to investigate their effects on SWB. The cross-country culture survey indices that originated in the study of organizational behavior have now been widely used, contributing to the formation of areas of studies such as cultural economics and cultural finance, as surveyed by Breuer and Quinten (2010). This provides a strong foundation for testing the relationships between culture and happiness more comprehensively. The earliest widely used cross-country culture survey indices are those of Hofstede (1980). Hofstede (1980) uses survey questionnaires on staff members in different branches of IBM in 53 different countries to calculate the culture indices of these countries. Four different dimensions are distinguished. For any dimension, each country has a corresponding culture value. Even for countries belonging to the same cultural area like Britain and the U.S., they have different culture values. The differences in culture in different countries no longer have to be reflected by the dummy variable of 0 and 1 but instead may be marked by more precise values. This significantly increases the discrimination ability of the cross-country comparison of cultural differences. There are mainly 4 items that measure the attitudes of residents in different countries towards issues that include individualism-collectivism, uncertainty, power distance, and gender equality (Hofstede 1980). Hofstede and Bond (1988) add a fifth dimension, long orientation, which measures the attitude of present-future orientation. Somewhat similar to the Hofstede (1980) survey of culture indices is the measurement of the 9 dimensions of culture by House et al. (2004) in the 1990s for 62 countries, forming the GLOBE culture indices. These indices repeat the four dimensions of Hofstede (1980) and add additional dimensions to examine the attitudes of residents in different countries towards assertiveness, performance, humane interaction, etc.

In this paper, we regress the GLOBE culture indices against the SWB levels of different countries to examine the explanatory power of culture indices on the country fixed effects in SWB. Our analysis involves five steps. First, we run an OLS regress between the traditional socio-economic, demographic variables and SWB in order to see their explanatory power on SWB. Secondly, we add country dummy variables to see whether there exist country fixed effects in SWB. Thirdly, we use culture indices to replace the country dummy variables in the previous step in order to discover the explanatory power of culture indices on the country fixed effects. Fourthly, we re-examine the ways culture may affect SWB. We do relative importance (RI) analysis to see which culture dimension affects SWB most, and compare the predicting power between culture variables and traditional variables such as income and social income comparison. Finally, we examine the issue of possible double directions of causality, or the problem of endogeneity (Markus and Kitayama 2010). For example, happier people may be more risk-averse and more willing to help others (Cahit 2009). To tackle this possible endogeneity problem, we use language dummies as instruments for the culture variables, and then run the GMM regression.

The contributions of this paper are as follows. First, we analyse the effects of culture on SWB from a wider perspective. Previous researchers have mainly used language or religion dummy variables to represent culture (e.g., Diener et al. 2003; Heukamp and Arino 2011). This only reflects some unidimensional characteristic of culture. The GLOBE culture indices used here include nine dimensions of culture characteristics. This allows us to analyse the influence of culture on happiness more comprehensively. Secondly, through the decomposition of the relative contribution to R^2^, we compare the relative importance of different culture factors.We find that, among the nine dimensions in GLOBE culture indices, Power Distance (PDI) and Gender Egalitarianism (GEI) play the most important and stable role in determining SWB. One implication is that, to raise the SWB of people, we may have to consider which culture dimensions are more important to improve. The empirical findings of this paper may provide some thoughts in this direction.

The rest of the paper is organized as follows. Section 2 discusses the relationships between culture and happiness, and draws out our hypotheses. Section 3 introduces our empirical model, methodology and data. Section 4 contains the empirical results. Section 5 concludes the whole paper.

## The Relationships Between National Cultural Indices and SWB

### Culture and SWB: Conceptual Analysis

Schyns (1998) points out that there are two theories that link culture and SWB. One is the “comparison theory”, according to which human happiness depends on comparisons between standards of quality of life and perceived life circumstances. The other is Maslow’s “needs theory”. According to Maslow, leading a good life will largely be determined by the extent of need-satisfaction. The more needs are satisfied, the happier people will be. The needs of people are classified into different levels. At the very basic level are the needs for survival, including food. Next, there are the physiological and safety needs, such as security, stability, dependency, freedom from fear and anxiety, structure, order, law and so on. After the satisfaction of the more basic needs, there will emerge the needs for love, affection and belonging. At an even higher level, there is the desire for esteem, reputation, prestige, fame and glory. Maslow also pointed out that higher need gratification produces more desirable subjective results, i.e. more profound happiness, serenity and richness of inner life (Maslow 1970).

The theory of comparison suggests that the satisfaction of material needs has limited contributions in increasing happiness. Even with increases in incomes, the effects on happiness may not be significant if the incomes of others also increase. According to the needs theory, basic needs such as material needs may be easily satisfied, but the effects of the satisfaction of these needs on happiness are limited, with diminishing marginal utility. On the other hand, satisfaction at the higher spiritual levels is limitless.^2^ The emphasis on the pursuit of material needs is nearly the same across different culture and different countries. But the emphasis on the spiritual pursuit differs across different cultures, making culture very important in affecting SWB.

Both theories suggest that economic or material satisfaction is relatively easily obtained. The effects of different cultures on this level of satisfaction do not differ much. Culture only has significant different effects on higher levels of need. For example, Eastern cultures put emphasis on interpersonal comparisons. While Euro-American cultures emphasize freedom and independence, Eastern cultures focus on harmony and collective interests, putting collectivism above individualism. We believe that both the comparison and the needs theories capture some important aspects affecting happiness.

### The Measurement of Culture

Various methods have been used to capture cultural characteristics. Two of the more widely used classifications are discussed below.

Hofstede and his co-authors classify aspects of national cultures into 5 dimensions based on the survey of 53 IBM branches across the world in the 1970s (Hofstede 1980; Hofstede and Bond 1988), namely, individualism, power distance, masculinity, uncertainty avoidance, and Confucian dynamism, to measure the attitudes of people towards groups, power, gender, uncertainty and the future.

The GLOBE culture project provides us with updated cultural values from surveys in 62 countries in the 1990s. The GLOBE project extends Hofstede’s 5 dimensions to 9 dimensions, including assertiveness orientation, institutional collectivism, in-group collectivism, future orientation, gender differentiation, humane orientation, performance orientation, power distance, and uncertainty avoidance. Among them, collectivism and individualism, power distance, future dimension, uncertainty avoidance, and gender egalitarianism are included in both classifications. In terms of individual/collectivism, GLOBE and Hofstede use opposite measures: Hofstede uses the dimension of individualism, while GLOBE uses collectivism, which is further divided into institutional collectivism and in-group collectivism. Gender differentiation in GLOBE is similar to the masculinity dimension in Hofstede’s system but also in the opposite direction. Humane orientation, performance orientation and assertiveness orientation are unique to the GLOBE system.

The GLOBE cultural indicators have two data sets, namely practice scores and value scores. The former is derived from the answers of the interviewees regarding the prevailing status of the country’s culture; the latter is about what the direction for the future should be. Since we mainly want to discuss the effects of culture on people’s perception of current happiness, following Chui and Kwok (2009), Tang and Koveos (2008), we use the GLOBE practice scores for testing the relationship between culture and happiness.

### The Influences of Culture on Happiness: Hypotheses Development

#### In-group Collectivism (ING)

This dimension refers to a closed, intense social organization, in which people are divided into the “in-group” and “outside group”; people expect care from others in the “in-group”, and they have high degrees of loyalty to the group in return. Consensus and collaborative efforts are regarded as more valuable than individual action.

Unlike the Hofstede culture indices, in the GLOBE culture indices system, there are two kinds of collectivism, namely In-group collectivism (ING) and institutional collectivism (INC). The former reflects the degree to which individuals take pride in their membership in small groups such as their families and circles of close friends (Javidan and House 2001).The latter refers to the extent to which societal institutions encourage the collective distribution of resources and collective action (House et al. 2002).

ING places more emphasis on the constraints on individuals and is more effective in a small group, and so it corresponds more to individualism in Hofstede’s indices, but in the opposite direction (Chui and Kwok 2008). In the empirical field, Arrindell et al. (1997) found that Hofstede’s individualism is significantly positively related to SWB. Therefore, ING may be negatively related to happiness. Thus we have the following hypothesis:

##### Hypothesis 1

The SWB of a country is negatively related to its level of In-group collectivism.

#### Institutional Collectivism (INC)

This dimension reflects the extent to which a society’s institutions favour collectivism versus autonomy (Javidan and House 2001). It also refers to the extent to which societal institutions encourage the collective distribution of resources and collective action (House et al. 2002).

Institutional collectivism is a little different from in-group collectivism in that it gives more emphasis to social support. Organizations in countries with high institutional collectivism tend to take more responsibility for the welfare of employees (Javidan and House 2001). Triandis et al. (1988) point out that when people face unpleasant life events, social and interpersonal support in collectivistic societies can help to buffer against stress and disease.

Diener et al. (1995) argue that the effects of individualism-collectivism on SWB cannot be predicted with certainty. In collectivistic countries, there might be greater feelings of social support, which ought to enhance SWB. In contrast, individualistic societies have more personal freedom, and individuals have a higher ability to pursue their individual goals, which also ought to enhance SWB. Since institutional collectivism gives more emphasis to social support comparing with in-group collectivism, we have the following hypothesis:

##### Hypothesis 2

The SWB of a country is positively related to its level of institutional collectivism.

#### Power Distance (PDI)

This dimension is the degree to which individuals expect and agree that power should be unequally shared (Javidan and House 2001). Hofstede argues that a large power distance in nations may lead to inequalities (between persons). Such inequalities could lead individuals to feel that they at the mercy of forces beyond their control. This causes greater externality, which is negatively correlated with mental well-being. A large power distance in nations goes hand in hand with inequalities, not only in work organizations but also in areas such as social status and prestige, wealth and civil rights. Thus a significantly negative association is predicted between PDI and national levels of SWB. Arrindell et al. (1997) also find a negative relationship between PDI and SWB. We therefore put forward our second hypothesis:

##### Hypothesis 3

The SWB of a country is negatively related to its level of PDI.

#### Gender Egalitarianism (GEI) 

This dimension refers the degree to which a society minimizes gender role differences (House et al. 2002). Higher GEI societies give women more space to control their life and may enhance the average SWB levels in the whole society. Arrindell et al. (1997) also argue that masculine societies are characterized by higher job stress and lower overall satisfaction at work, and feminine nations have significantly higher average SWB scores. Barnett and Baruch (1987) discover that in a society with a higher GEI, women have more choices in social roles, leading to their higher self-assessed health levels, such as lower rates of sickness and lower usage of drugs, hence contributing to a higher SWB. One would expect the feminine (higher GEI) nations to have significantly higher average scores on SWB than the more masculine ones. So we have hypothesized as follows:

##### Hypothesis 4

The SWB of a country is positively related to its level of GEI.

#### Uncertainty Avoidance (UAI)

This dimension is defined as a country’s reliance on social norms and procedures to alleviate uncertain future events (Javidan and House 2001). In Hofstede’s view, societies with high UAI scores are typified by nervousness, lower ambition for individual advancement, and pessimism about work-related issues. Using Hofstede’s culture UAI data, Arrindell et al. (1997) found that UAI is negatively related to SWB. But in the GLOBE culture index system, as Chui and Kwok (2008) pointed out, UAI mainly refers to the extent to which individuals in a country seek orderliness, consistent structured lifestyles, a clear specification of social expectations, and rules and laws to cover unpredictable situations (Javidan and House 2001). Therefore, a high UAI may not necessary lead to stress and nervousness. One specific question asked in the GLOGE survey regarding this dimension is: “Most people lead highly structured lives with few unexpected events” (House et al. 2004). People in high UAI countries tend to agree with this statement. This indicates that people in high uncertainty avoidance societies have fewer unpredictable future events. According to Maslow’s needs theory, the need for safety is the most important basic level need after survival. The satisfaction of the safety need ensures stable and long-term SWB. UAI may approximately reflect the effort in the pursuit of the safety need. Thus we have the following hypothesis:

##### Hypothesis 5

The SWB of a country is positively related to its level of UAI.

#### Assertiveness Orientation (AOI)

This dimension refers to the degree to which individuals are encouraged to be tough and competitive (Javidan and House 2001). Javidan and House (2001) also pointed out that people in highly assertive societies tend to value competition and sympathize with the strong. Chui and Kwok (2008) indicated that people in highly assertive societies are less inclined to help others when they are in need. This means that people may easily feel helpless when they suffer difficulties, which may decrease their SWB. The excessive focus on competition results in tensions and pressures. It is also related to comparison. According to the theory of social comparison, the excessive competition of social comparison may lead to only one victor and many failures, reducing the general level of SWB. So we put forward the following hypothesis:

##### Hypothesis 6

The SWB of a country is negatively related to its level of AOI.

#### Future Orientation (FOI)

This dimension refers to the extent to which a society encourages planning, investing in the future, and delaying gratification (Javidan and House 2001). Gratification in the future is an important part of people’s overall happiness. Emphasis on the future can increase control over future uncertainty. The need for security is very important in Maslow’s theory, as it contributes to basic satisfaction in the long run. The emphasis on the future contributes to the avoidance of uncertainty and provides a basic level of satisfaction with life. We thus have the following hypothesis:

##### Hypothesis 7

SWB is positively related to the level of future orientation.

#### Humane Orientation (HOI)

This dimension refers to the degree to which a society encourages and rewards individuals for being fair, altruistic, generous, caring and kind to others (Javidan and House 2001). People in societies with a high humane orientation are expected to care for the needs of others, and the social security network in countries with high HOI is likely to be stronger than in countries with a low humane orientation. This means that individuals will not be too unhappy when they have difficulties. Moreover, according to Maslow’s theory, after the satisfaction of lower levels needs and subject to ability levels, generosity and helping others are higher level needs that may provide larger contributions towards SWB. Being kind to others makes individuals feel good (the warm glow effect). Thus we have the following hypothesis:

##### Hypothesis 8

SWB is positively related to the level of humane orientation.

#### Performance Orientation (POI)

This dimension reflects the extent to which a community encourages and rewards innovation, high standards and performance improvement (House et al. 2004). McClelland (1987) introduces the concept of need for achievement, which is defined as the need to do better. With this need people tend to achieve pleasure from progressive improvement. In a high performance orientation country, improvement and innovation will be rewarded, leading to higher SWB at the individual level. Improvement and innovation also contribute to self-esteem and self-realization, which are higher level needs in Maslow’s theory and very important for SWB. The dimension of Performance is also closer to Individualism. Generally, a more individualist society also encourages competition and Performance more. We put forward our hypothesis as follows (Table 1):

**Table 1:** Hypothesized effects of GLOBE’s cultural practices on SWB

Cultural practices	Hypothesized effects
In-group collectivism(ING)	−
Institutional collectivism(INC)	+
Power distance(PDI)	−
Gender egalitarianism (GEI)	+
Uncertainty avoidance(UAI)	+
Assertiveness (AOI)	−
Future orientation(FOI)	+
Humane orientation(HOI)	+
Performance orientation(POI)	+

##### Hypothesis 9

SWB is positively related to the level of Performance orientation.

## Model, Data and Empirical Design

### Model and Variables

In order to test the hypotheses in Sect. 2, we regress SWB on various culture dimensions. We set up our regression equation as follows:

1 $$ {\text{SWB}}_{it} = \beta_{0} + \beta_{1} \;{\text{Culture}}_{i} + \beta_{2} \;{\text{Controls}}_{it} + \varepsilon_{it} $$

where the dependent variable SWB_*it*_ is the subjective well-being level for country *i* in year *t*. The explanatory variables (Culture_*i*_) are a set of cultural indices, which measure different dimensions of culture. Controls are variables that may affect happiness, and *ε*_*it*_ is the disturbance term.

The SWB indices are provided by the World Values Survey, which have been used widely in the literature. Over the past few decades, the World Values Surveys have interviewed representative national samples of scores of countries, with an average sample size of 1,400 respondents in a country. Over 1,000 publications have been based on these data.

There are three categories of variables related to happiness in the World Values Survey, namely happiness, life satisfaction and SWB. Life satisfaction was assessed by asking respondents to indicate how satisfied they were with their life as a whole, using a scale that ranged from 1 (not at all satisfied) to 10 (very satisfied). Happiness was assessed by asking respondents to indicate how happy they were, using four categories: very happy, rather happy, not very happy, and not at all happy. Inglehart et al. (2008) combine these two variables into one—subjective well-being (SWB). Life satisfaction is measured on a 10-point scale and happiness is measured on a 4-point scale, and because the two questions have opposite polarity, the SWB composite was constructed as follows: SWB = life satisfaction − 2.5*happiness. If 100 % of its people were very happy and extremely satisfied, a country would get the maximum score of 7.5. If happiness and life satisfaction were evenly balanced, the country would get a score of zero. If 100 % of its people were not at all happy and extremely unsatisfied, the country would score –9. Life satisfaction represents the cognitive part of happiness, reflecting people’s cognitive view on the various areas of life. Happiness reflects the sentimental part of happy like delight, satisfaction and other passive sentiments (Andrews and Mckennell 1980). Inglehart et al. (2008) pointed out that SWB contains two parts, and it may be better than any one single variable.

The explanatory variables are cultural indices. We mainly take the widely used GLOBE set of cultural indices including the nine dimensions (see Sect. 2.2 for details). We also use the Hofstede culture indices for robustness check.

As there are many factors affecting happiness, we must control for these factors to overcome the bias of omitted variables. Following the steps outlined in previous literature (Arrindell et al. 1997; Diener et al. 1995), we select national income (GDP), growth of income (GDPRATE), human rights (RIGHTS), population density (POP), education (EDU), and social comparison of income (SCI) as control variables. National income is proxied by GDP, which represents the income level. In general, income level is related positively to SWB (see Diener et al. 1993; Veenhoven 1993). Growth of income was proxied by the growth rate of GDP. According to Diener et al. (1993), a rapid rise in income would lead to lower SWB because rapid growth would probably be accompanied by high aspirations, as well as by dislocations such as employment moves and family separation. Education (EDU) is measured by the enrollment ratio in tertiary education of each nation, which is predicted to be positively related with SWB. Human rights (RIGHTS) refers to the condition of a country in protecting an individual’s civil and political rights. Since freedom and security are good for people, this variable is predicted to be positively related with SWB (Diener et al. 1995). Population density is the percentage of the population of each country living in urban areas of one million or more individuals (World Development Report). A higher population density may mean a poorer quality of life and hence lowered SWB. On the other hand, higher density may be related to a higher provision of public amenities and contribute to SWB. According to the comparison theory, comparison of income can predict SWB better than income itself and is negatively related with SWB, but Arrindell et al. (1997) find that SCI is positively related with SWB.

### Data

The data on SWB come from the World Values Survey (Inglehart et al. 2008). Our explanatory variable (culture_*i*_) comes from House et al. (2004) and Hofstede and Bond (1988). Control variables mainly come from Arrindell et al. (1997). We use a panel data set of 48 countries over the years from 1990 to 2006 to estimate Eq. (1). The definitions and data sources of key variables are detailed in Table 2. The descriptive statistics of variables are presented in Table 3.

**Table 2:** Definitions and sources of variables

Variables	Definitions and sources
SWB	Subjective well-being, referring to how people experience the quality of their lives and it includes both emotional reactions and cognitive judgments
	*Source*: Inglehart et al. (2008)
GLOBE cultural indices	The future orientation index (FOI), uncertainty avoidance index (UAI), institutional collectivism (INC), in-group collectivism (ING), assertiveness (AOI), power distance (PDI), performance (POI), humane (HOI), gender egalitarianism (GEI)
	*Source*: House et al. (2004)
Hofstede cultural indices	The long-term orientation (H-Long), uncertainty avoidance index (H-UAI), individualism index (H-IND), power distance (H-PDI), masculinity(H-MAS) *Source*: Hofstede and Bond (1988)
GDP	GDP in constant 2000 US 100-billion dollars
	*Source*: *WBDD*
GDP growth	The growth rate of GDP
	*Source*: *WBDD*
Human rights (RIGHTS)	The condition of a country in protecting individual’s civil or political rights
	*Source*: Arrindell et al. 1997
Population density (POP)	The percentage of the population of each nation living in urban areas
	*Source*: Arrindell et al. 1997
Social comparison of income (SCI)	The log of mean value of a nation’s bordering nations’ gross domestic product per capita.
	*Source*: Arrindell et al. 1997
Education (EDU)	Enrollment ratio in tertiary education
	*Source*: *WBDD*
Language dummies	Five 0/1 variables (English, Chinese, Spanish, French, and Dutch) used to indicate the official language of each country
	*Source*: Stulz and Williamson (2003)

**Table 3:** Descriptive statistics

Variables	Mean	SD	Min	Max	N
Subjective well-being (SWB)	1.90	1.75	–2.78	4.48	842
GDP (GDP)	70.10	1,470	3.88	1,330	804
GDP growth rate (GDPRATE)	2.56	5.25	–32.12	14.20	833
Education (EDU)	43.01	20.68	2.90	97.98	746
Human rights (RIGHTS)	6.18	2.39	4.41	11.62	493
Population density (POP)	24.45	14.98	0.00	61.00	493
Social comparison of income (SCI)	8.94	1.25	5.53	10.11	493
In-group collectivism (ING)	4.89	0.75	3.46	5.86	581
Institutional collectivism (INC)	4.31	0.45	3.63	5.26	581
Power distance (PDI)	5.21	0.35	4.14	5.70	581
Gender egalitarianism (GEI)	3.40	0.37	2.45	4.07	581
Uncertainty avoidance (UAI)	4.30	0.64	3.09	5.42	581
Assertiveness orientation (AOI)	4.20	0.35	3.41	4.77	581
Future orientation (FOI)	3.91	0.46	3.06	4.80	581
Humane orientation (HOI)	3.94	0.39	3.29	4.96	581
Performance orientation (POI)	4.13	0.37	3.50	5.04	581
Individualism (H-IND)	6.02	2.18	1.30	9.10	442
The long-term orientation (H-Long)	4.39	1.98	2.30	8.00	204
Masculinity(H-MAS)	5.29	2.09	0.50	9.50	442
Power distance (H-PDI)	4.60	1.84	1.10	8.10	442
Uncertainty avoidance (H-UAI)	6.25	2.12	2.30	10.4	442

The definitions and sources of variables are presented in Table 2

Table 4 presents the correlation matrix of key variables. It is shown that, among the traditional economic variables, the SWB is significantly positively related to education (EDU), and the mean income of a nation’s bordering nations (SCI), and negatively related to GDP growth rate (GDPRATE), human rights (RIGHTS), and population density (POP). SWB also significantly related to culture indices. We find that SWB is positively related to INC, GEI, UAI, FOI, HOI, and POI, and negatively related to PDI and AOI.

**Table 4:** Correlation matrix

	SWB	GDP	GDPRATE	EDU	RIGHTS	POP	SCI	ING	INC	PDI	GEI	UAI	AOI	FOI	HOI
GDP (GDP)	0.05														
GDP growth rate (GDPRATE)	–0.09	–0.01													
Education (EDU)	0.29*	0.15*	–0.04												
Human rights (RIGHTS)	–0.42*	–0.03	0.24*	–0.61*											
Population density (POP)	–0.16*	0.26*	–0.06	0.20*	0.01										
Social comparison of income (SCI)	0.53*	–0.08	–0.26*	0.51*	–0.76*	–0.20*									
In-group Collectivism (ING)	–0.50*	0.00	0.26*	–0.46*	0.65*	0.10*	–0.57*								
Institutional Collectivism (INC)	0.15*	–0.14*	0.07	0.36*	–0.18*	–0.08	–0.03	–0.44*							
Power Distance (PDI)	–0.60*	0.02	0.06	–0.19*	0.27*	0.13*	–0.25*	0.60*	–0.40*						
Gender Egalitarianism (GEI)	0.50*	–0.06	–0.26*	0.13*	–0.37*	–0.09	0.51*	–0.46*	–0.03	–0.48*					
Uncertainty Avoidance (UAI)	0.43*	–0.18*	–0.24*	0.31*	–0.68*	–0.21*	0.67*	–0.78*	0.23*	–0.39*	0.35*				
Assertiveness (AOI)	–0.10*	0.15*	–0.09	–0.07	0.10*	0.34*	0.08	0.08	–0.53*	0.14*	–0.18*	0.08			
Future orientation (FOI)	0.30*	–0.04	–0.12*	0.20*	–0.42*	–0.02	0.26*	–0.59*	0.38*	–0.60*	0.19*	0.68*	0.17*		
Humane orientation (HOI)	0.38*	–0.02	0.21*	0.23*	–0.20*	–0.24*	0.05	–0.21*	0.51*	–0.56*	0.18*	0.06	–0.62*	0.20*	
Performance orientation (POI)	0.35*	0.02	0.00	0.28*	–0.33*	–0.09	0.17*	–0.44*	0.27*	–0.27*	–0.20*	0.42*	0.27*	0.43*	0.18*

This table presents the Pearson correlation coefficients between variables. * denotes significant at the 5 % level or better

Given the fact that our model contains so many variables, possible serious multi-collinearity is an issue that has to be considered. Note that, the correlation coefficient of control variables (e.g. GDP-SCI) and culture indices are not too high. In most cases, the correlation coefficients are less than 0.8. In later regression analysis, we calculate the variance inflation factors (VIF) of the various variables. The mean values of VIF in all models are less than 10, and the VIF of each variable in the model are less than 5. This indicates that we can safely ignore the problem of multi-collinearity and use these variables to regress. Due to space limitation, the results for VIF are not reported in the paper; interested readers may email us for them.

### Methodology

We try two kinds of regression. Firstly, we run OLS tests to get the empirical relationships between SWB and culture. Secondly, we do GMM estimation using language dummies as instruments to tackle the problem of possible endogeneity.

We do the OLS regressions in three steps. In the first step, we run an OLS regress between SWB and national income, growth of income, human rights, population density, education, and social comparison of income to test the power of traditional variables in explaining SWB. In the second step, we add country dummy variables into the regression in order to investigate whether there exist fixed effects which cannot be explained by the traditional variables. In the third step, we replace country dummy variables with cultural variables in the regression to test the effects of cultural variables on SWB.

The possible double directions of causality may exist between culture and happiness, which induces the endogeneity problem. Markus and Kitayama (2010) point out that, culture and the self are mutually determined. Happiness is a major experience of the self. Thus, though culture may have important effects on happiness, happiness may also affect cultural affiliation. For example, happier people value life more, and are more willing to maintain the current situation and hence less willing to take risks. Their degrees of risk aversion are thus likely higher. The survey of 313,354 subjects by Goudie et al. (2010) shows that, people with higher SWB are more willing to wear safety belts while driving. Cahit (2009) also points out that, SWB is important in influencing risk aversion and financial behaviour. Happier people are more conscious of risks and more willing to choose safe investment. Also, happier people have better future expectation and longer life expectation (Cahit 2009; Veenhoven 2008); people scoring high on Future and with higher SWB are more generous (Kirchsteiger et al. 2006), and more willing to respect social order in their behavior (Lyubomirsky et al. 2005), and hence may score higher in the dimension of Humane.

To deal with the potential endogeneity problem, we further perform GMM regression between SWB and culture variables. We take the language dummy variables as instruments because language dummies are closely related to culture but not necessarily related to SWB. In some studies of cross-culture comparative research, language dummies are usually used as proxies or instruments of culture (Diener et al. 2003; Stulz and Williamson 2003). Language data are taken from Stulz and Williamson (2003).

## Empirical Results

### OLS Results

OLS regression results are shown in Table 5. Column (1) shows that among traditional explanatory variables, Income and Social Comparison of Income (SCI) are significantly positively related to happiness. The coefficients of GDP growth rate (GDPRATE), Education (EDU) and Human rights (RIGHTS) are positive but not significant. These are basically the same as Arrindell et al. (1997). We can see that the R^2^ of the regression is low at the level of 0.30. Column (2) shows the result of regression with country dummy variables, where R^2^ sharply increases to 0.90, which implies that there exist significant country fixed effects in the SWB differences across countries.

**Table 5:** OLS regression

	(1)	(2)	(3)	(4)	(5)	(6)	(7)	(8)	(9)	(10)	(11)	(12)	(13)
GDP (GDP)	0.061*** (3.68)	0.009 (0.60)	0.061*** (3.83)	0.089*** (4.85)	0.062*** (3.94)	0.061*** (4.54)	0.073*** (4.11)	0.072*** (4.23)	0.068*** (3.91)	0.065*** (4.08)	0.062*** (3.62)	0.082*** (4.91)	0.081*** (5.02)
GDP growth rate (GDPRATE)	1.918 (1.10)	–0.783 (–0.77)	3.642** (2.07)	2.022 (1.16)	2.066 (1.25)	3.458** (2.05)	2.246 (1.27)	1.807 (1.06)	2.204 (1.22)	–1.198 (–0.68)	1.652 (0.96)	1.674 (1.13)	1.995 (1.33)
Education (EDU)	0.048 (0.23)	–0.016 (–0.15)	–0.218 (–1.17)	–0.607*** (–2.81)	–0.172 (–1.09)	0.373* (1.92)	0.134 (0.65)	–0.142 (–0.68)	0.090 (0.43)	–0.592*** (–3.30)	–0.226 (–1.04)	–0.185 (–0.81)	–0.111 (–0.47)
Human rights (RIGHTS)	–0.022 (–0.56)	–0.164* (–1.68)	0.046 (1.09)	0.011 (0.26)	0.020 (0.54)	–0.019 (–0.54)	0.017 (0.40)	0.009 (0.22)	0.032 (0.78)	0.052 (1.27)	0.033 (0.87)	0.142*** (3.81)	0.143*** (3.90)
Population density (POP)	–0.652** (–2.03)	0.553 (1.05)	–0.386 (–1.25)	–0.219 (–0.68)	–0.175 (–0.70)	–0.794** (–2.54)	–0.549 (–1.60)	–0.114 (–0.31)	–0.619* (–1.85)	0.466 (1.60)	–0.306 (–0.97)	1.230*** (4.04)	1.092*** (3.47)
Social comparison of income (SCI)	0.418*** (7.21)	0.115 (0.85)	0.389*** (6.61)	0.539*** (8.95)	0.400*** (7.65)	0.251*** (4.20)	0.377*** (6.27)	0.500*** (7.92)	0.448*** (7.69)	0.567*** (9.88)	0.484*** (8.64)	0.531*** (7.23)	0.493*** (6.22)
In–group collectivism (ING)			–0.473*** (–6.42)									0.376*** (3.79)	0.232* (1.78)
Institutional collectivism (INC)				0.531*** (5.54)								–0.352*** (–2.70)	–0.460*** (–3.20)
Power distance (PDI)					–1.407*** (–20.06)							–1.359*** (–9.45)	–1.265*** (–7.96)
Gender Egalitarianism (GEI)						1.109*** (7.21)						0.741*** (4.69)	0.710*** (4.58)
Uncertainty avoidance (UAI)							0.285*** (2.81)					0.182 (1.40)	0.112 (0.85)
Assertiveness (AOI)								–0.483*** (–3.65)				–1.372*** (–5.56)	–1.328*** (–5.39)
Future orientation (FOI)									0.526*** (5.12)			–0.085 (–0.53)	–0.079 (–0.51)
Humane orientation (HOI)										1.064*** (15.10)		–0.237 (–1.50)	–0.074 (–0.40)
Performance orientation (POI)											0.866*** (9.76)	1.692*** (10.30)	1.692*** (10.60)
Constant	–0.809 (–1.12)	2.859 (1.57)	1.259 (1.52)	–4.253*** (–4.53)	6.341*** (8.36)	–3.213*** (–4.19)	–2.038** (–2.36)	0.233 (0.31)	–3.581*** (–4.15)	–6.721*** (–7.78)	–5.347*** (–6.46)	0.202 (0.11)	0.793 (0.43)
Country dummies	No	Yes	No	No	No	No	No	No	No	No	No	No	No
Language dummies	No	No	No	No	No	No	No	No	No	No	No	No	Yes
N	442	442	442	442	442	442	442	442	442	442	442	442	442
R^2^	0.304	0.898	0.363	0.340	0.526	0.387	0.316	0.323	0.336	0.439	0.368	0.654	0.656
adj–R^2^	0.294	0.891	0.353	0.329	0.518	0.377	0.305	0.312	0.325	0.430	0.358	0.641	0.643

This table reports the results of the pooled OLS regressions of the SWB on future (FOI), uncertainty avoidance (UAI), institutional collectivism (INC), in–group collectivism (ING), assertiveness orientation (AOI), gender egalitarianism (GEI), humane orientation (HOI), performance orientation (POI), power distance (PDI), and some control variables. The robust t statistics are in parentheses

***, **, * Significance at 1, 5, and 10 %, respectively

The country fixed effects do not change over time, and cannot be explained by traditional variables like income. To explore the details of these fixed effects, we add cultural dimensions into the regression equation. The results are shown in Columns (3) to (11).

Column (5) shows that PDI is significantly negatively related to SWB, consistent with our hypothesis 3 and Arrindell et al. (1997). As for R^2^, we find a great increase, from 0.30 to 0.53. This contribution to R^2^ is the highest among all culture variables. This means that, out of the nine culture variables, PDI is most powerful in explaining differences in SWB. It also means that culture is a very important predicting factor of SWB levels across countries.

As discussed in Sect. 2 above, collectivism/individualism is regarded as being one of the most important culture dimensions affecting happiness. collectivism is divided into two dimensions, one being ING, and the other INC. Column (3) shows that ING is significantly negatively related to SWB. This is consistent with Diener et al. (1995) and Arrindell et al. (1997) since ING is said to be directly contrary to individualism in the Hofstede culture indices (Chui and Kwok 2008). Column (4) shows that INC is significantly positively related to SWB. These two results are consistent with our hypothesis 1 and hypothesis 2. This means that there exist big differences between the two collectivism indices in the GLOBE culture indices. Our explanation is that INC is different from ING in that it puts more emphasis on social networks and social help, and hence it is positively related to SWB.

Columns (6) to (12) show that Gender Egalitarianism (GEI), Uncertainty Avoidance (UAI), Performance Orientation (POI), and Future Orientation (FOI) are significantly positively related to SWB. Assertiveness (AOI) is significantly negatively related to SWB. These results are all consistent with our hypotheses 4–9 and also consistent with Diener et al. (1995) and Arrindell et al. (1997). The coefficient of UAI is significantly positive, which is consistent with our hypothesis 5 but inconsistent with Arrindell et al. (1997). Our explanation is that UAI in the GLOBE culture indices is not the same as risk aversion, and it is also different from UAI in the Hofstede culture indices which are used in Arrindell et al. (1997). It measures the degree of order in life and therefore is positively related to SWB.

### Relative Importance Analysis

As our focus is the different effects of different culture characteristics on SWB, we are more concerned with the relative importance of the nine culture indices. In other words, we wish to isolate the contribution of each explanatory variable towards the R^2^ or adjusted R^2^ of the whole model. To achieve this, we use the method of Relative Importance (RI) analysis that has been widely used recently in management, psychology, and sociology.^3^ The basic idea of RI is to compare the relative importance of different explanatory variables after model formation.

RI is concerned with rank ordering the predictors in terms of relative importance by comparing the additional contributions the predictors make to the variance reproduced (or explained) by all possible subset models (consisting of subsets of the predictors). The additional contribution of a predictor is measured as the increase in explained variance, or the increase in R^2^ (the variance accounted for by the model), when the predictor is added to a given subset model.

Obviously, if there does not exist any correlation between all explanatory variables, we only have to calculate the covariance of each explanatory variable with the explained variable, and divide it with the variance of the explained variable to obtain the degree of contribution of that variable. However, in most regressions, significant correlations exist between variables. For our case here, as may be seen from the coefficients of correlation in Table 4, most coefficients of correlation between most variables are significant at the 5 % level. In such cases, we have to consider the correlation to evaluate the contribution of a variable towards the R^2^.

The RI of a variable x is defined as the additional contributions (AC) of *x* towards R^2^. In calculating the AC of a variable *x*, we have to consider all possible degrees of contribution of *x* in all subset models under the original model. For example, consider a model with only two explanatory variables (*x*_1_ and *x*_2_), i.e., *y* = *a* + *β*_1_ *x*_1_ + *β*_2_ *x*_2_ + *ε*. Using R^2^(*x*_1_, *x*_2_) for the goodness of fit of the model, there are two ways to represent the contribution of *x*_2_ to *y*. One is to consider the subset model: *y* = *a* +*β*_2_ *x*_2_ + *ε*, where only *x*_2_ is included. The contribution of *x*_2_ here is RI_1_ = R^2^ (*x*_2_). Another way is adding *x*_2_ to the subset model *y* = *a* + *β*_1_ *x*_1_ + *ε* to get the model *y* = *a* + *β*_1_ *x*_1_ + *β*_2_ *x*_2_ + *ε.* Then the contribution of *x*_2_ here is RI_2_ = R^2^(*x*_1_, *x*_2_) - R^2^(*x*_1_).

Obviously, in the process above, if the correlation coefficient between *x*_1_ and *x*_2_ is not zero as usually the case, RI_1_ tends to overestimate the contribution of *x*_2_ and RI_2_ tends to underestimate. Thus, Budescu (1993) and Azen and Budescu (2003) use the average value of the two estimates, i.e. RI = (RI_1_ + RI_2_)/2 as the contribution of *x*_2_.

In the example above, the model only includes two explanatory variables and the calculation is relatively simple. In most analyses, the model usually includes many explanatory variables. Using k for the number of explanatory variables in the model, the original model corresponds to 2 ^*k*^-1 subset models. For example, with *k* = 5, there are 31 subset models. Regression has to be done on all subset models to calculate the relative contribution of each variable and using the average value as the final calculated degree of contribution of the variable.^4^

To facilitate analysis, we use the method of Krasikova et al. (2011) and Tonidandel and LeBreton (2011) to standardize the values of RI reported below. Specifically, the RI of all variables is aggregated into RI total. Then the ratio of the RI of each variable to RI total is calculated to get the standardized degree of contribution. This standardization has the advantage of making the sum of the standardized degrees of contribution of all explanatory variables equal to one, making the relative importance of each variable becomes easily comparable with others.

Table 6 presents the results of relative importance (RI) analysis. As our focus is on the effects of culture variables on SWB, in Table 6, we just present the RI results of column (3)-(12) in Table 5. In Table 5, given the model specification in column (3), the R^2^ of the model is 0.363. The corresponding RI results shown in column (3) of Table 6 show that, the most importance determinants of SWB in this specification is SCI and ING, with RI values 39.4 % and 31.7 %, respectively. Columns (4)-(11) in Table 6 present the RI of the remaining eight culture variables.

**Table 6:** Relative importance (RI) analysis

	(3)	(4)	(5)	(6)	(7)	(8)	(9)	(10)	(11)	(12)
GDP (GDP)	1.8 % [6]	3.0 % [6]	1.3 % [6]	1.9 % [6]	3.0 % [6]	2.5 % [6]	2.3 % [6]	1.6 % [6]	1.8 % [6]	
GDP growth rate (GDPRATE)	1.4 % [7]	1.1 % [7]	0.6 % [7]	1.4 % [7]	1.1 % [7]	1.2 % [7]	1.1 % [7]	1.2 % [7]	1.0 % [7]	
Education (EDU)	6.3 % [4]	8.8 % [3]	4.7 % [4]	7.3 % [4]	7.9 % [4]	9.1 % [3]	8.1 % [4]	6.3 % [4]	7.1 % [4]	9.2 % [4]
Human rights (RIGHTS)	15.4 % [3]	21.2 % [2]	11.7 % [3]	15.4 % [3]	17.6 % [3]	22.5 % [2]	19.3 % [2]	15.7 % [3]	17.9 % [3]	
Population density (POP)	4.1 % [5]	5.0 % [5]	2.6 % [5]	4.6 % [5]	4.8 % [5]	4.9 % [4]	5.5 % [5]	3.1 % [5]	4.3 % [5]	
Social comparison of income (SCI)	39.4 % [1]	54.3 % [1]	29.0 % [2]	32.4 % [2]	45.0 % [1]	55.7 % [1]	50.1 % [1]	43.6 % [1]	47.6 % [1]	8.7 % [5]
In-group collectivism (ING)	31.7 % [2]									8.5 % [6]
Institutional collectivism (INC)		6.6 % [4]								1.7 % [10]
Power distance (PDI)			50.0 % [1]							23.6 % [1]
Gender Egalitarianism (GEI)				37.1 % [1]						17.2 % [2]
Uncertainty Avoidance (UAI)					20.7 % [2]					6.1 % [8]
Assertiveness (AOI)						4.0 % [5]				1.6 % [11]
Future orientation (FOI)							13.6 % [3]			3.9 % [9]
Humane orientation (HOI)								28.4 % [2]		8.1 % [7]
Performance orientation (POI)									20.3 % [2]	11.4 % [3]
Combinations	127	127	127	127	127	127	127	127	127	2,047

(1) Column 1 in the table shows the indicators of Relative Importance (RI) of each variable. We first break down the model R-squared into shares from individual regressors and, the RI of the *j* th variable is its share in explaining the dependent variable variance. See Grömping (2007) for details. The relative ranking of each variable is presented in brackets. (2) In column (12), 9.2 % denotes the RI of a set of variables, including GDP, GDPRATE, EDU, RIGHTS and POP

The results in Table 6 show that, among all culture variables, PDI and GEI have the highest RI in the contribution to explaining the R^2^, reaching 50 % and 37 % respectively. This is consistent with our OLS of Table 5. At the same time, results reported in column 3 of Table 6 show that, after adding PDI to the regression equation, the RI of this variable reaches 50 %, which equals the sum of 6 other traditional explanatory variables. This demonstrates that the explanatory power on SWB of this culture factor (PDI) far exceeds traditional explanatory variables. Among the 9 culture variables, there are 6 variables where the contribution to explaining the R^2^ of their own respective equation exceeds 20 %. In the regression equation (Eq 12) that include all the cultural and traditional variables, the three variables PDI, GEI, POI each has RI of more than 10 %, surpassing the sum (9.2 %) of those of the six traditional variables. The RI each of ING, INC, HOI exceeds 8 %, close to the sum (9.2 %) of those of the six traditional variables. These results show that these culture variables have strong predictive power of SWB.

### Endogeneity Issues

As mentioned in Sect. 3.3 on methodology, there may exist endogeneity problems between culture and SWB (Cahit 2009; Markus and Kitayama 2010; Goudie et al. 2010). For example, it may be the case that the happier people have higher degrees of uncertainty avoidance, are more generous and have longer life expectation. To tackle this possible endogeneity problem, we do a GMM test between culture and happiness.

We choose language dummies as instrument variables. Language dummies are significantly related to culture and are often used as a proxy variable in the culture research literature (Stulz and Williamson 2003). The relationship is not so obvious between language and happiness. We cannot conclude that some people feel happier just because he can speak a particular language. For this reason we think that language dummies are good instrument variables for culture. The data for the Language variables come from Stulz and Williamson (2003).

The results of the GMM regression are shown in Table 7.^5^ We find that the results for ING, PDI, FOI, GOI, HOI, POI, and AOI are the same as our bench test. The sign of the coefficients of the INC and UAI variables are still positive, but not significant. In general, the results of the GMM test are basically the same as the benchmark OLS test.

**Table 7:** National culture and SWB (GMM estimation)

	(1)	(2)	(3)	(4)	(5)	(6)	(7)	(8)	(9)
GDP (GDP)	0.044*** (2.90)	0.071*** (4.70)	0.058*** (4.71)	0.055*** (4.70)	0.064*** (4.46)	0.083*** (5.42)	0.075*** (5.41)	0.071*** (5.25)	0.067*** (4.67)
GDP growth rate (GDPRATE)	5.057*** (2.95)	3.429** (2.06)	4.503*** (2.92)	6.408*** (3.78)	1.446 (0.82)	3.053* (1.85)	3.908** (2.32)	2.367 (1.40)	2.836* (1.72)
Education (EDU)	−0.477*** (−2.61)	−0.156 (−0.64)	−0.072 (−0.46)	0.289 (1.34)	−0.346* (−1.71)	−0.278 (−1.37)	−0.149 (−0.75)	−0.371** (−2.01)	−0.394** (−1.99)
Human rights (RIGHTS)	−0.014 (−0.39)	−0.031 (−0.89)	−0.027 (−0.82)	−0.049 (−1.46)	−0.067* (−1.74)	−0.024 (−0.60)	0.009 (0.25)	−0.026 (−0.75)	−0.023 (−0.71)
Population density (POP)	−0.204 (−0.71)	−0.718*** (−2.59)	−0.192 (−0.78)	−0.790*** (−2.84)	−0.976*** (−3.38)	−0.655* (−1.88)	−0.759*** (−2.60)	−0.371 (−1.19)	−0.540* (−1.95)
Social comparison of income (SCI)	0.459*** (8.60)	0.522*** (8.33)	0.422*** (9.03)	0.260*** (3.60)	0.578*** (10.34)	0.527*** (7.62)	0.557*** (11.15)	0.540*** (10.20)	0.559*** (11.36)
In-group collectivism (ING)	−0.434*** (−4.50)								
Institutional collectivism (INC)		−0.086 (−0.73)							
Power distance (PDI)			−0.987*** (−12.74)						
Gender egalitarianism (GEI)				1.204*** (4.52)					
Uncertainty avoidance (UAI)					−0.366** (−2.48)				
Assertiveness (AOI)						−0.081 (−0.37)			
Future orientation (FOI)							0.420*** (2.87)		
Humane orientation (HOI)								0.281* (1.69)	
Performance orientation (POI)									0.427** (2.15)
Constant	0.838 (0.99)	−1.218 (−1.15)	4.108*** (5.83)	−3.430*** (−4.20)	0.003 (0.80)	−1.304* (−1.76)	−3.898*** (−3.86)	−2.863*** (−2.64)	−3.722*** (−3.33)
N	442	442	442	442	442	442	442	442	442
R^2^	0.340	0.274	0.494	0.376	0.238	0.297	0.316	0.352	0.330
adj-R^2^	0.329	0.263	0.486	0.366	0.225	0.286	0.305	0.341	0.319

This table reports the results of the GMM regressions of the SWB on future (FOI), uncertainty avoidance (UAI), institutional collectivism (INC), in-group collectivism (ING), assertiveness orientation (AOI), gender egalitarianism (GEI), humane orientation (HOI), performance orientation (POI), power distance (PDI), and some control variables. The robust t statistics are in parentheses

***, **, * Significance at 1, 5, and 10 %, respectively

### Robustness Test

The focus of this paper is on culture and SWB. Empirically, there are many different measures of these two variables. Thus, there may exist measurement errors. On the side of culture, there are two widely used indices: the GLOBE indices and the earlier Hofstede indices. This paper mainly uses the former which have the advantages of having more dimensions with more up-to-date data. In this sub-section, we further use the Hofstede culture indices (H-index) to replace the GLOBE indices to test for robustness. For the measurement of happiness, there are three indices in WVS, namely happiness, life satisfaction and SWB. In our main tests, we use SWB. In the robustness test, we use happiness and life satisfaction in the regression.^6^

Table 8 reports results of the regression using the Hofstede culture indices. We use OLS and GMM separately, getting basically consistent results for both tests. For the control variables, GDP and SCI are significantly positive coefficients and very stable. The coefficient for population density is significantly negative but not stable. GDPRATE, EDU, and RIGHTS do not have significant effects in most of the time. For the GMM test of 5 culture variables, H-IND and H-Long are significantly related to SWB positively; H-PDI, and H-MAS (opposite to the feminine index GEI in the GLOBE culture indices system) are significantly negatively related to SWB. These results are all consistent with those using the GLOBE indices reported in Table 6. In the OLS test, the signs of the coefficients of H-IND, H-MAS are consistent with the GMM test, but not significant. In particular, the coefficients of H-UAI in GMM and OLS tests are significantly negative. This is opposite to the coefficient of UAI for GLOBE in Table 6. This is mainly because UAI has quite different meanings in the Hofstede and the GLOBE indices, as discussed under hypothesis 4. Also, Hofstede (2001, p.148) warns that “uncertainty avoidance does not equal risk avoidance.” Chui and Kwok (2008, 2009) show that the effect of Hofstede’s Uncertainty Avoidance is inconsistent with people’s usual intuition when they regress insurance and culture, and the GLOBE’s Uncertainty Avoidance index is more consistent with our intuition. The GLOBE cultural practice indices have another advantage in that that they are more up to date and have more dimensions than Hofstede’s cultural indices. So in this paper we rely on the results of the GLOBE practical cultural indices to report the relationship between culture and SWB. Perhaps we need more cultural indices on risk avoidance in the future to get more confident conclusions.

**Table 8:** National culture and SWB (Hofstede culture index)

	(1)	(2)	(3)	(4)	(5)	(6)	(7)	(8)	(9)	(10)
	OLS					GMM				
GDP (GDP)	0.061*** (3.14)	0.163*** (9.31)	0.067*** (3.82)	0.072*** (4.49)	0.050*** (2.99)	0.039** (2.24)	0.163*** (12.27)	0.101*** (5.95)	0.057*** (3.80)	0.031** (2.05)
GDP growth rate (GDPRATE)	1.919 (1.10)	−3.497* (−1.84)	1.899 (1.08)	1.714 (0.98)	0.454 (0.26)	3.324* (1.94)	−3.676** (−2.12)	3.616** (2.14)	3.121* (1.90)	2.540 (1.61)
Education (EDU)	0.045 (0.22)	−0.270 (−1.47)	−0.002 (−0.01)	−0.256 (−1.29)	−0.194 (−1.06)	−0.295 (−1.60)	−0.390*** (−3.42)	−0.519*** (−2.59)	−0.582*** (−3.09)	−0.378** (−2.27)
Human rights (RIGHTS)	−0.021 (−0.56)	0.541*** (7.40)	−0.023 (−0.59)	0.032 (0.78)	0.023 (0.60)	−0.020 (−0.56)	0.505*** (9.17)	−0.033 (−0.96)	−0.022 (−0.63)	−0.052 (−1.58)
Population density (POP)	−0.648* (−1.94)	−3.840*** (−9.62)	−0.631* (−1.96)	−0.417 (−1.32)	0.103 (0.34)	−0.278 (−1.00)	−3.791*** (−15.85)	−0.600** (−2.04)	−0.274 (−0.99)	0.256 (0.89)
Social comparison of income (SCI)	0.417*** (6.98)	1.288*** (12.07)	0.416*** (7.12)	0.373*** (6.06)	0.452*** (7.73)	0.464*** (8.18)	1.253*** (17.23)	0.525*** (10.44)	0.404*** (6.69)	0.468*** (9.14)
H-IND	0.002 (0.06)					0.089*** (3.67)				
H-Long		0.291*** (7.06)					0.246*** (10.68)			
H-MAS			−0.016 (−0.94)					−0.071*** (−3.92)		
H-PDI				−0.169*** (−6.20)					−0.217*** (−4.56)	
H-UAI					−0.167*** (−11.97)					−0.126*** (−5.87)
Constant	−0.821 (−1.15)	−11.897*** (−8.41)	−0.691 (−0.91)	0.112 (0.14)	−0.375 (−0.50)	−1.700*** (−2.64)	−11.145*** (−11.67)	−1.143* (−1.76)	0.432 (0.53)	−0.303 (−0.45)
N	442	204	442	442	442	442	204	442	442	442
R^2^	0.304	0.640	0.305	0.349	0.397	0.272	0.632	0.279	0.319	0.373
adj-R^2^	0.293	0.627	0.293	0.339	0.387	0.260	0.619	0.268	0.308	0.363

The t statistics are in parentheses

***, **, * Significance at 1, 5, and 10 %, respectively

The test results using happiness and life satisfaction are largely consistent with those using SWB. They are not reported here to save space.

## Conclusions

Cross-country differences in subjective well-being (SWB) are an important issue of high interest. The existing literature has discussed this widely from different perspectives. As economic and demographic factors, including income levels, have been found to be of limited explanatory power for the cross-country differences in SWB, culture is regarded as a possible factor that accounts for the differences in the mean levels of SWB. This paper uses the GLOBE culture indices and the World Values Survey SWB data to investigate the relationship between culture and SWB. Our main purpose is to compare the explanatory power of culture variables relative to traditional factors, and the relative importance of different culture variables in explaining the differences in SWB between countries.

Our empirical results show that the traditional economic and demographic factors have low explanatory power over the cross-country differences in SWB, with a regression R^2^ of only 0.30. The addition of country dummy variables increases the R^2^ enormously to 0.90. This shows the existence of very significant country fixed effects. Empirically, these country fixed effects are related to stable, time-invariant national characteristics like cultural, geographical and climatic factors. As we replace the country dummy variables with cultural variables like PDI, the value of R^2^ also increases significantly from 0.30 to 0.53. This suggests that culture may be the main factor for country fixed effects. Our empirical results show that culture variables have significant effects in the regression on SWB, suggesting that culture is an important explanatory variable for SWB. To explore the explanatory power on SWB of different culture dimensions, we undertake RI analysis. We discover that, in a regression on SWB together with traditional variables like GDP, and the culture variables, the contribution to R^2^ of the culture variable PDI is as high as 50 %. This equals the sum of six traditional variables including income. Other culture variables like GEI, INC, HOI are also more important than the traditional variables. Putting all control variables and culture variables into the regression, the combined contribution of the 6 traditional variables including income is only 9.2 %, while the combined contribution of culture variables accounts for 91.8 %. These results show that culture is a very strong predicting factor of SWB.

Our results have significant implications. Our empirical results show that while GDP has significant and positive correlation with SWB, it explains only 3 % of the variation in SWB between countries, far less than culture variables. Among the 9 culture dimensions, our results show that PDI and GEI are most significant and stable. Thus, to increase SWB, emphasis on these two culture factors may be desirable. As PDI reflects power distance and correlates significantly negatively with SWB, decreasing power distance and strengthening democracy may contribute positively to SWB, GEI measures gender balance and correlates significantly positively with WEB. This suggests that raising gender equality may also be important for SWB. However, the specific ways how PDI may be reduced and GEI increased without significant costs and other undesirable side effects is beyond the scope of this paper.

There are some inadequacies in the present study. First, the data for culture variables and for SWB are from two different surveys. This may cause some divergences. Secondly, our empirical results show that Social Comparison of Income (SCI) is significantly positively related with SWB. Though this result is consistent with Arrindell et al. (1997), it is not consistent with our intuition. One possible explanation may be that people in many countries do not compare with those in neighbouring countries but with reference countries. For example, people in Singapore may compare more with people in reference countries/regions like Hong Kong, Taiwan, and Korea (as they belong to the four tigers and have similar cultural backgrounds and closer income levels) instead of the neighbouring countries of Malaysia and Indonesia which have different cultures and much lower income levels. Thirdly, we only discuss the importance of culture on SWB but have not analysed the channels or mechanisms of the relevant effects. Further studies on these may be needed.

Looking to the future, the following studies may be desirable. First, we hope that the WVS will simultaneously include questions on SWB and on more cultural dimensions,^7^ or do surveys on both culture factors and SWB for at least some of the countries. This may reduce measurement errors for future research. Secondly, for SCI, some ways of identifying the relevant reference countries may be used, instead of just using the geographically neighboring countries. Thirdly, cross multiplication of culture variables with traditional variables of income, population, education, etc. may be used to identify how culture variables affect happiness through its effects on the various micro or macro variables. Fourthly, many emerging and transitional countries like China, Russia, and Vietnam have undergone drastic economic-systemic transformation and fast economic growth; how these changes may affect the relationships between culture and happiness is also worth exploring.

## Footnotes

1 Arrindell et al. (1997) is an exception. They use the Hofstede culture indices to explain happiness.

2 We take this as figuratively speaking, not literally.

3 The analysis of relative contribution has been described as Relative Importance or as Dominance Analysis. Johnson and LeBreton (2004), Grömping (2007), Fortin et al. (2011), Krasikova et al. (2011), Nathans et al. (2012), Luo and Azen (2013), Nimon and Oswald (2013) have very detailed discussion of this.

4 The Stata command “domin” is used for RI analysis in this paper.

5 The results of first stage regression show that the culture variables are highly related to the instruments, implying that there is no weak instruments problem. Limited to the space, the first stage results are not presented, but can be provided upon request.

6 The results of robust check are similar to those reported in the paper when SWB is used. Limited to the space, these results are not presented, but can be provided upon request.

7 At present, WVS only has questions on culture-related factors such as thrift, trust, and obedience, but not on many important culture variables like individualism/collectivism, power distance, uncertainty avoidance, and future orientation.

## References

[CR1] Andrews F, Mckennell A (1980). Measures of self-reported well-being: Their affective, cognitive and other components. Social Indicators Research.

[CR2] Arrindell WA, Hatzichristou C, Wensink J, Rosenberg E, van Twillert B, Stedema J, Meijer D (1997). Dimensions of national culture as predictors of cross-national differences in subjective well-being. Personality and Individual Differences.

[CR3] Azen R, Budescu DV (2003). The dominance analysis approach for comparing predictors in multiple regression. Psychological Methods.

[CR4] Barnett RC, Baruch GK, Barnett RC, Biener L, Baruch GK (1987). Social roles, gender, and psychological distress. Gender and stress.

[CR5] Benson C (2000). The cultural psychology of self: Place, morality and art in human worlds.

[CR6] Breuer W. & Quinten B. (2010). *Cultural finance*. SSRN working paper.

[CR7] Bruner J (1990). Acts of meaning.

[CR8] Budescu DV (1993). Dominance analysis: A new approach to the problem of relative importance of predictors in multiple regression. Psychological Bulletin.

[CR9] Cahit, G. (2009). *Are happier people better citizens*? German Socio-Economic Panel Study (SOEP) working paper.

[CR10] Chui A, Kwok C (2008). National culture and life insurance consumption. Journal of International Business Studies.

[CR11] Chui A, Kwok C (2009). Cultural practices and life insurance consumption: An international analysis using GLOBE score. Journal of Multinational Financial Management.

[CR13] Diener E (2000). Subjective well-being. The science of happiness and a proposal for a national index. American Psychologist.

[CR14] Diener E, Diener M, Diener C (1995). Factors predicting the subjective well-being of nations. Journal of Personality and Social Psychology.

[CR15] Diener E, Kahneman D, Helliwell J (2010). International differences in well-being.

[CR16] Diener E, Oishi S, Richard E, Lucas R (2003). Personality, culture, and subjective well-being: Emotional and cognitive evaluations of life. Annual Review of Psychology.

[CR58] Diener, E., Sandvik, E., Seidlitz, L. & Diener, M. (1993). The relationship between income and subjective well-being: Relative or absolute? *Social Indicators Research, 28*(3), 195–223.

[CR17] Fortin N, Lemieux T, Firpo S, Orley A, David C (2011). Chapter 1—Decomposition methods in economics. Handbook of labor economics.

[CR18] Goudie, R. J., Mukherjee, S., De Neve, J.-E., Oswald, A. J., & Wu, S. (2010). *Happiness as a driver of risk*-*avoiding behavior*. CESIFO working paper no. 3451.

[CR19] Grömping U (2007). Estimators of relative importance in linear regression based on variance decomposition. The American Statistician.

[CR20] Guiso L, Sapienza P, Zingales L (2006). Does culture affect economic outcomes?. Journal of Economic Perspectives.

[CR21] Heukamp FH, Arino MA (2011). Does country matter for subjective well-being?. Social Indicators Research.

[CR22] Hofstede G (1980). Culture’s consequences: International differences in work-related values.

[CR23] Hofstede G (2001). Culture’s consequences: Comparing values, behaviors, institutions, and organizations across nations.

[CR24] Hofstede G, Bond MH (1988). The confucius connection: From cultural roots to economic growth. Organizational Dynamics.

[CR25] House, R. J., Hanges, P. J., Javidan, M., Dorfman, P. W., & Gupta, V. (2004). *Culture, leadership, and organizations: The GLOBE study of 62 societies*. Thousand Oaks.

[CR26] House R, Javidan M, Hanges P, Dorfman P (2002). Understanding cultures and implicit leadership theories across the GLOBE: An introduction to project GLOBE. Journal of World Business.

[CR27] Inglehart R, Foa R, Peterson C, Welzel C (2008). Development, freedom and rising happiness: A global perspective (1981–2007). Perspectives on Psychological Science.

[CR28] Inglehart R, Klingemann H-D, Diener E, Suh M (2000). Genes, culture, democracy, and happiness. Culture and subjective well-being.

[CR29] Javidan M, House RJ (2001). Cultural acumen for the global manager: Lessons from project GLOBE. Organizational Dynamics.

[CR30] Johnson JW, LeBreton JM (2004). History and use of relative importance indices in organizational research. Organizational Research Methods.

[CR31] Kenny C (1999). Does growth cause happiness, or does happiness cause growth?. Kyklos.

[CR59] Kirchsteiger, G., Rigotti, L. & Rustichini, A. (2006). Your morals might be your moods. *Journal of Economic Behavior & Organization, 59*(2), 155–172.

[CR32] Kitayama, S. (2002). Culture and basic psychological processes—Toward a system view of culture: Comment on Oyserman et al. (2002). *Psychological Bulletin, 128*(1), 89–96. 10.1037/0033-2909.128.1.89 11843550

[CR33] Krasikova D, LeBreton JM, Tonidandel S (2011). Estimating the relative importance of variables in multiple regression models. International Review of Industrial and Organizational Psychology.

[CR34] Kwan VSY, Bond M, Singelis TM (1997). Pan cultural explanations for life-satisfaction: Adding relationship harmony to self-esteem. Journal of Personality and Social Psychology.

[CR35] Layard R (2005). Happiness: Lessons from a new science.

[CR36] Lu L, Gilmour R (2004). Culture and conceptions of happiness: Individual oriented and social oriented SWB. Journal of Happiness Studies.

[CR37] Lu L, Gilmour R, Kao SF (2001). Cultural values and happiness: An east–west dialogue. Journal of Social Psychology.

[CR38] Luo W, Azen R (2013). Determining predictor importance in hierarchical linear models using dominance analysis. Journal of Educational and Behavioral Statistics.

[CR60] Lyubomirsky S, King L, Diener E (2005). The benefits of frequent positive affect: Does happiness lead to success?. Psychological Bulletin.

[CR39] Markus HR, Kitayama S (1991). Culture and the self: Implications for cognition, emotion, and motivation. Psychological Review.

[CR40] Markus HR, Kitayama S (2010). Cultures and selves: A cycle of mutual constitution. Perspectives on Psychological Science.

[CR41] Maslow AH (1970). Motivation and personality.

[CR42] McClelland, D. C. (1987). Human motivation. *CUP Archive.*

[CR43] Nathans LL, Oswald FL, Nimon K (2012). Interpreting multiple linear regression: A guidebook of variable importance. Practical Assessment, Research & Evaluation.

[CR44] Ng Y-K (2002). The East-Asian happiness gap. Pacific Economic Review.

[CR45] Nimon KF, Oswald FL (2013). Understanding the results of multiple linear regression: Beyond standardized regression coefficients. Organizational Research Methods.

[CR46] Oishi S, Diener E, Lucas RE (1999). Cross-cultural variations in predictors of life satisfaction: Perspectives from needs and values. Personality and Social Psychology Bulletin.

[CR47] Schimmack U, Oishi S, Diener E (2002). Cultural influences on the relation between pleasant emotions and unpleasant emotions: Asian dialectic philosophies or individualism-collectivism?. Cognition and Emotion.

[CR48] Schyns P (1998). Cross national differences in happiness: Economic and cultural factors explored. Social Indicators Research.

[CR49] Shweder RA, Sullivan MA (1993). Cultural psychology: Who needs it?. Annual Review of Psychology.

[CR50] Stulz RM, Williamson R (2003). Culture, openness, and finance. Journal of Financial Economics.

[CR51] Suh EM (2002). Culture, identity consistency, and subjective well-being. Journal of Personality and Social Psychology.

[CR52] Tang L, Koveos P (2008). A framework to update Hofstede’s cultural value indices: Economic dynamics and institutional stability. Journal of International Business Studies.

[CR53] Tonidandel S, LeBreton JM (2011). Relative importance analysis: A useful supplement to regression analysis. Journal of Business and Psychology.

[CR54] Triandis HC, Bontempo R, Villareal MJ, Asai M, Lucca N (1988). Individualism and collectivism: Cross-cultural perspective on self-in group relationships. Journal of Personality and Social Psychology.

[CR55] Uchida Y, Norasakkunkit V, Kitayama S (2004). Cultural constructions of happiness: Theory and empirical evidence. Journal of Happiness Studies.

[CR56] Veenhoven R (1993). Happiness in nations.

[CR57] Veenhoven R (2008). Healthy happiness: Effects of happiness on physical health and the consequences for preventive health care. Journal of Happiness Studies.

